# Modeling convection-diffusion-reaction systems for microfluidic molecular communications with surface-based receivers in Internet of Bio-Nano Things

**DOI:** 10.1371/journal.pone.0192202

**Published:** 2018-02-07

**Authors:** Murat Kuscu, Ozgur B. Akan

**Affiliations:** 1 Internet of Everything (IoE) Group, Electrical Engineering Division, Department of Engineering, University of Cambridge, Cambridge, CB3 0FA, United Kingdom; 2 Next-generation and Wireless Communications Laboratory (NWCL), Department of Electrical and Electronics Engineering, Koc University, Istanbul, 34450, Turkey; North University of China, CHINA

## Abstract

We consider a microfluidic molecular communication (MC) system, where the concentration-encoded molecular messages are transported via fluid flow-induced convection and diffusion, and detected by a surface-based MC receiver with ligand receptors placed at the bottom of the microfluidic channel. The overall system is a convection-diffusion-reaction system that can only be solved by numerical methods, e.g., finite element analysis (FEA). However, analytical models are key for the information and communication technology (ICT), as they enable an optimisation framework to develop advanced communication techniques, such as optimum detection methods and reliable transmission schemes. In this direction, we develop an analytical model to approximate the expected time course of bound receptor concentration, i.e., the received signal used to decode the transmitted messages. The model obviates the need for computationally expensive numerical methods by capturing the nonlinearities caused by laminar flow resulting in parabolic velocity profile, and finite number of ligand receptors leading to receiver saturation. The model also captures the effects of reactive surface depletion layer resulting from the mass transport limitations and moving reaction boundary originated from the passage of finite-duration molecular concentration pulse over the receiver surface. Based on the proposed model, we derive closed form analytical expressions that approximate the received pulse width, pulse delay and pulse amplitude, which can be used to optimize the system from an ICT perspective. We evaluate the accuracy of the proposed model by comparing model-based analytical results to the numerical results obtained by solving the exact system model with COMSOL Multiphysics.

## Introduction

Internet of Bio-Nano Things (IoBNT) is an emerging technology defining the seamless connection of nanomachines and biological entities with each other and with conventional macroscale networks, such as the Internet [1, 2]. It has an enormous potential to transform the way we connect with and understand the world “at the bottom”, by extending the human consciousness and control with bio-nano things collaboratively sensing and acting upon the environments never explored by any other paradigm before. To enable the IoBNT and its groundbreaking applications, such as continuous health monitoring and smart drug delivery, it is imperative to devise artificial communication techniques at nanoscale and bio-cyber interfaces to connect bio-nano things with the macroscale networks. Bio-inspired molecular communications, where molecules are used to encode, transmit and receive information, stands as the most promising technique to enable nanonetworks, since it is intrinsically biocompatible, energy efficient, and reliable in confined geometries, where conventional techniques like electromagnetic communication does not properly work. There is a significant body of work in MC literature, including channel models, modulation schemes and communication protocols, designed and tailored to the peculiarities of nanoscale and molecular physics and limited capabilities of bio-nano things. [3–7].

In this paper, we investigate a particular type of MC system, where the concentration-encoded molecular information is conveyed via diffusion and convection induced by fluid flow to a surface-based reactive receiver in a microfluidic channel. The receiver containing ligand receptors on its surface is placed at the bottom wall of the channel and samples the propagating information molecules, i.e., ligands, based on ligand-receptor binding reaction. The concentration of bound receptors is informative of the transmitted ligand concentration, thus, used to decode the transmitted message. After the passage of the ligand plug, the channel and receiver return to their initial states due to the clearance by the continuous fluid flow. End-to-end system can be defined as a *convection-diffusion-reaction system*, which is highly nonlinear; and the finite duration of transmitted pulses makes the problem even more nonlinear and complex.

Convection-diffusion-reaction systems are especially prominent in microfluidic sensing and chromatography applications, such as affinity chromatography [8], microfluidic surface plasmon resonance (SPR) sensing [9], where analytes are propagated over ligand-specific receptor assays. In addition to the studies targeting microfluidic surface-based biosensing technologies, such as planar thin gold film SPR sensors [10], semiconductor bioFETs [11], a considerable amount of efforts has been devoted to modeling and control of the complex interplay between convection, diffusion and reaction to optimize the efficiency of analyte transport [12, 13].

Molecular transport in microfluidic channels has been recently addressed from communication theoretical perspective in [14, 15], which develop end-to-end channel models for the linear time-invariant systems consisting of biological transmitters and receivers placed in chambers along the microfluidic channel. Moreover, the response of bacterial receivers within microfluidic channels are experimentally reported in [16], where empirical models based on linear approximations are developed for the transient response of bacteria to pulse-amplitude-modulated molecular messages. Additionally, digital microfluidic MC networks based on droplets have been studied in [17]. Accounting for the surface-based receivers, our study targets more complicated systems that are neither linear nor time-invariant.

Microfluidic MC channels integrated with surface-based molecular receivers is promising for groundbreaking applications within the IoBNT framework. For example, in an *in vivo* continuous health monitoring application, mobile nanosensors circulating within cardiovascular system can inform a bio-cyber gateway placed at the interior surface of blood vessels about their sensing operations through molecular signals in blood flow, where convection and diffusion act simultaneously on the transport of molecules [1]. Furthermore, it can also find use in microfluidic networked lab-on-a-chip devices, which is an emerging technology to diversify the point-of-care medical applications and increase the efficiency of on-chip diagnostics [18]. Moreover, imitating the transport of molecules with convection and diffusion in confined geometries like vascular and neuro-synaptic channels, similar microfluidic configurations can find application in organs-on-chips and artificial synapses relying on molecular information and communication technologies [3, 19–21].

The overall process, which covers the release of ligands by the transmitter in the form of a finite-duration concentration pulse, molecular transport in laminar flow through microfluidic channel, and the molecular detection by the surface receiver equipped with finite number of ligand receptors is a highly nonlinear and time-varying process, and does not yield an analytical solution for the ligand and bound receptor concentration fields. Therefore, it necessitates the application of computationally-expensive numerical methods, like finite element analysis (FEA). In this study, we develop an end-to-end analytical model that can capture the expected time course of the received signal in terms of number of bound receptors. The model is based on quasi-steady state two-compartment model, which we tailor to the time-varying characteristics of the microfluidic MC system. The resulting model captures the effect of channel and receiver geometry, and system parameters regarding the fluid flow and ligand-receptor reaction. It accounts for the nonlinearities caused by laminar flow resulting in parabolic velocity profile and finite number of receptors resulting in saturation of the receiver. The effect of interplay between reaction and transport rates, which can lead to depletion layer over the receiver surface is also covered. Based on the developed model, we derive approximate analytical expressions for the received pulse delay, pulse amplitude and pulse width to help characterize and optimize the system from communication theoretical perspective. The analytical results are compared to numerical solutions obtained with the exact system model by using COMSOL Multiphysics, which is a finite element analysis simulation software.

The remainder of the paper is organized as follows. In the Methods section, we present the exact model of the communication system with nonlinear partial differential equations, and we introduce our approximate model and derive the communication theoretical metrics for the received signal. A comparative analysis of the analytical and numerical results is provided in the Results and Discussions section. Finally, the concluding remarks are given at the end of the paper.

## Methods

### Communication system model

**Fig 1:**
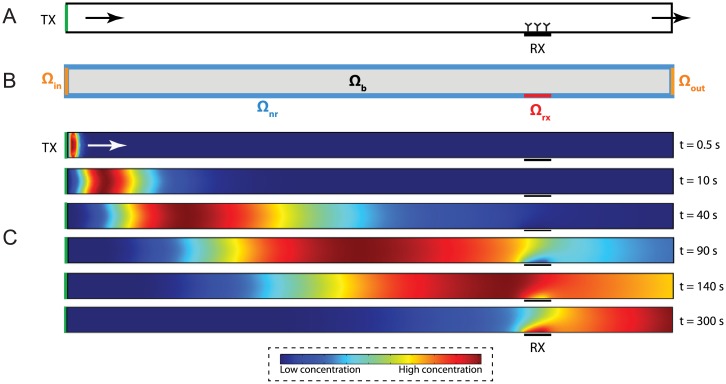
Microfluidic MC with surface-based receiver. (A) Conceptual drawing of the system. (B) Domains and boundaries used in the exact system model. (C) Demonstration of concentration-encoded molecular message propagation through the reactive surface of receiver in convection-diffusion channel. Finite element simulations are carried out in COMSOL Multiphysics.

In this section, we represent the end-to-end model of the microfluidic communication channel as a system of partial differential equations. We utilize a two-dimensional (2-d) model considering a microfluidic channel with rectangular cross section as shown in Fig 1A. 2-d models are proved effective in modeling the molecular transport, especially when there is an obvious interplay between convection, diffusion and surface reaction, as the uniformity of the molecular concentration along y-direction is disturbed above the reactive surface [22]. On the other hand, for cases, where there is no reactive surface, 1-d models can succesfully capture the effect of convection and diffusion [23].

Using a similar notation to that of [22], we define three orthogonal domains: (i) bulk domain **Ω**_**b**_, where the convection and diffusion of ligands occur, (ii) reacting surface domain **Ω**_**rx**_ denoting the biorecognition layer of the receiver, where the ligand-receptor reaction occurs, and (iii) non-reacting surface domains **Ω**_**nr**_ defining the walls of the microfluidic channel excluding the receiver surface, as demonstrated in Fig 1B. We also define two dependent variables *c* = *c*(*x*, *y*, *t*) denoting the ligand concentration in space and time domain, and *R* = *R*(*x*, *t*) denoting the bound receptor concentration at the receiver surface.

Hence, in a 2-d convection-diffusion system the propagation of ligands is governed by

$$\begin{array}{c} \frac{\partial c}{\partial t}=D\nabla^{2}c-u_{x}\left(y\right)\frac{\partial c}{\partial x},\;\left(x,y\right)\in \Omega_{b}, \end{array}$$ (1)

where $\nabla^{2}=\frac{\partial^{2}}{\partial x^{2}}+\frac{\partial^{2}}{\partial y^{2}}$ is the two dimensional Laplace operator, *u*_*x*_(*y*) is the flow velocity as a function of distance to the surrounding walls. We assume fully developed laminar flow in the microfluidic channel yielding a parabolic flow velocity profile, i.e.,

$$\begin{array}{c} u_{x}\left(y\right)=4u\left(y/h\right)\left(1-y/h\right),\;\left(x,y\right)\in \Omega_{b}, \end{array}$$ (2)

where *u* is the maximum flow velocity. *D* is the effective diffusion coefficient taking into account the effect of Taylor-Aris type dispersion [14]. For a channel with rectangular cross-section, it is given by

$$\begin{array}{c} D=\left(1+\frac{8.5u^{2}h_{ch}^{2}w_{ch}^{2}}{210D_{0}^{2}\left(h_{ch}^{2}+2.4h_{ch}w_{ch}+w_{ch}^{2}\right)}\right)D_{0}, \end{array}$$ (3)

where the intrinsic diffusion coefficient is denoted by *D*_0_ [14]. Here, *h*_*ch*_ and *w*_*ch*_ denote the height and width of the microfluidic channel, respectively.

No-flux boundary condition is assigned to the non-reacting walls of the channel, i.e.,

$$\begin{array}{c} \frac{\partial c}{\partial y}=0,\;y\in \Omega_{\text{nr}}. \end{array}$$ (4)

Flux condition at the reactive boundary, where ligand-receptor reaction occurs is given by

$$\begin{array}{c} -D\frac{\partial c}{\partial y}=R\left(R,c\right),\;y\in \Omega_{\text{rx}}, \end{array}$$ (5)

where *R* is the bound receptor concentration, and R denotes the reactive flux.

We define the inlet and outlet boundary conditions as

$$\begin{array}{c} c\left(x=0,y,t\right)=c_{in}\left(t\right), \end{array}$$ (6)

$$\begin{array}{c} \frac{\partial c(x=L,y,t)}{\partial x}=0, \end{array}$$ (7)

where *c*_*in*_(*t*) is the transmitted signal.

Assuming no surface diffusion for receptors at the receiver surface, bound receptor concentration as a function of time can be written as

$$\begin{array}{c} \frac{\partial R}{\partial t}=R\left(R,c\right),\;\left(x,y=0\right)\in \Omega_{\text{rx}}. \end{array}$$ (8)

Given the finite receptor concentration at the receiver, ligand-receptor binding reaction can be described by the first-order Langmuir kinetics giving the reactive flux:

$$\begin{array}{c} R\left(R,c\right)={k_{1}c|}_{y=0}\left(R_{max}-R\right)-k_{-1}R,\;\left(x,y=0\right)\in \Omega_{\text{rx}}. \end{array}$$ (9)

Lastly, the initial conditions for the system are defined as

$$\begin{array}{c} R\left(x,0\right)=0,\;\left(x,y=0\right)\in \Omega_{\text{rx}}. \end{array}$$ (10)

$$\begin{array}{c} c\left(x,y,0\right)=0,\;\left(x,y\right)\in \Omega_{b}. \end{array}$$ (11)

The exact system model presented above is not analytically tractable and necessitates numerical methods to compute the ligand and bound receptor concentration.

### Proposed model

We develop an analytical model that approximates the time course of the mean number of bound receptors on the receiver surface for a rectangular ligand concentration pulse transmitted at the channel inlet, as shown in Fig 1C. Prior to modeling, it is worth elaborating briefly on the impact of conditions resulting from the competition between ligand transport and ligand-receptor reaction. In convection-diffusion-reaction systems, if the convective/diffusive transport in the channel supply ligands much more quickly than the receptors can bind them, then the system becomes reaction-limited, implying that transport dynamics has negligible effect on the resulting waveform for the bound receptor concentration. In such cases, ligand concentration near the receiver surface can be assumed equal to the concentration of ligands supplied at the channel inlet, and the well-mixed condition can be assumed for modeling the ligand-receptor reaction [22].

In practical systems, however, the reaction-limited condition often does not hold, because the concentration of supplied ligands is not continuous, giving rise to a concentration gradient between the channel inlet and the reactive surface [24, 25]. Furthermore, in microfluidic channels, the fluid flow is usually laminar leading to a parabolic flow velocity profile above the reactive surface, implying a concentration gradient from the center of the channel toward the reactive surface [22]. Moreover, when the system is utilized for communication purposes, reaction rates at the receiver surface should be kept large enough compared to the transport rate in order not to cause intersymbol interference (ISI). Because of these reasons, we assume that the system would be operating either in transport-limited regime, where the reaction rates are much larger than the ligand transport rate, or under partial mass transport limitations, caused by transport and reaction rates comparable to each other. In such cases, mass transport limitations could have substantial impact on the time course of bound receptor concentration, and well-mixed condition for the surface reactions often does not hold.

A compartmental approach is developed in [26] to model the ligand-receptor kinetics affected by mass transport limitation. It is based on dividing the space domain into two compartments, in each of which the ligand concentration can be assumed steady. This steady-state assumption proved to be effective in describing the isolated association and dissociation phases (two-stage process). The two-compartment model is widely employed in BIAcore analyses to determine the affinity of various ligand-receptor pairs [24].

We make use of the two-compartment model by tailoring it to the peculiarities of the system under investigation. We will demonstrate that this simple yet effective strategy can be used to reveal the characteristics of the microfluidic MC channel.

#### Two-Compartment model

Here, we review the basics of two-compartment model that we modify to propose an approximate model for the microfluidic communication channel.

In the two-compartment model, ligand concentration of the first compartment is assumed to be equal to the concentration at the channel inlet *c* = *c*_0_ [26]. The concentration in the second compartment covering the receptors *c*|_*y* = 0_ could be different than the bulk concentration due to the binding reactions occurring at the receiver surface. Concentration gradient between two compartments results in a flux of ligands expressed by

$$\begin{array}{c} J=k_{T}\left(c_{0}-{c|}_{y=0}\right), \end{array}$$ (12)

where *k*_*T*_ is the mass transport parameter, which is a function of the channel and receiver geometries, and the system parameters regarding diffusion and convection of ligands [25], i.e.,

$$\begin{array}{c} k_{T}=C_{T}A_{rx}\sqrt[{3}]{\frac{D^{2}F}{h_{ch}^{2}w_{ch}\left(d_{rx}+l_{rx}\right)}}, \end{array}$$ (13)

where *A*_*rx*_ = *l*_*rx*_*w*_*rx*_ is the receiver surface area with the receiver width *w*_*rx*_ = *w*_*ch*_. Here, *C*_*T*_ is defined as

$$\begin{array}{c} C_{T}=1.47\left(\frac{1-{\left(d_{rx}/\left(d_{rx}+l_{rx}\right)\right)}^{2/3}}{1-d_{rx}/\left(d_{rx}+l_{rx}\right)}\right), \end{array}$$ (14)

where *d*_*rx*_ is the minimum distance of the channel inlet to the receiver, *l*_*rx*_ is the length of receiver along the x-axis, and *F* = *h*_*ch*_ × *w*_*ch*_ × *u* is the maximum flow rate.

The steady-state solution of this two-compartment model for ligand concentration near the receiver surface is obtained by equating the ligand flux to the surface J given in Eq (12) to the reactive flux R given by Eq (9), i.e.,

$$\begin{array}{c} {c|}_{y=0}=\frac{k_{T}c_{0}+k_{-1}N_{R}}{k_{1}\left(N_{R,max}-N_{R}\right)+k_{T}}, \end{array}$$ (15)

where the position dependent surface receptor concentrations in Eq (9) are integrated over the receiver surface to obtain the number of receptors in units of *mol*, i.e., *N*_*R*,*max*_ = *R*_*max*_ *A*_*rx*_, *N*_*R*_ = ∫_*S*_ *R* *dA*_*rx*_.

Substituting this expression into Eq (9) yields a nonlinear differential equation representing the evolution of the number of bound receptors, i.e.,

$$\begin{array}{c} \frac{dN_{R}}{dt}=\frac{k_{1}k_{T}c_{0}\left(N_{R,max}-N_{R}\right)-k_{-1}k_{T}N_{R}}{k_{1}\left(N_{R,max}-N_{R}\right)+k_{T}} \end{array}$$ (16)

The solution of Eq (16) for the association phase is then obtained by setting the initial conditions as *c*(*x*, *y*, 0) = *c*_0_ and *R*(0) = 0 [25, 27], i.e.,

$$\begin{array}{c} N_{R,a}\left(t\right)=N_{R,eq}\left(1-\frac{W_{0}\left(\alpha -\beta t\right)}{\alpha }\right), \end{array}$$ (17)

where

$$\begin{array}{c} N_{R,eq}=\frac{c_{0}}{c_{0}+K_{D}}N_{R,max}, \end{array}$$ (18)

$$\begin{array}{c} \alpha =\frac{k_{1}c_{0}N_{R,max}}{k_{-1}N_{R,max}+k_{T}\left(c_{0}+K_{D}\right)}, \end{array}$$ (19)

$$\begin{array}{c} \beta =\frac{k_{1}c_{0}+k_{-1}}{1+\frac{k_{-1}N_{R,max}}{k_{T}\left(c_{0}+K_{D}\right)}}. \end{array}$$ (20)

The solution of Eq (16) for the dissociation phase can be found by setting the initial conditions as *c*(*x*, *y*, 0) = 0 and *N*_*R*_(0) = *N*_*R*,0_, with *N*_*R*,0_ being the number of bound receptors at the start of the dissociation phase [25, 27], i.e.,

$$\begin{array}{c} N_{R,d}\left(t\right)=\gamma W_{0}\left(-\frac{N_{R,0}}{\gamma }\exp\left(\frac{-k_{1}N_{R,0}-k_{T}k_{-1}\left(t-t_{0}\right)}{k_{1}\gamma }\right)\right), \end{array}$$ (21)

where

$$\begin{array}{c} \gamma =\frac{k_{1}N_{R,max}+k_{T}}{k_{1}}. \end{array}$$ (22)

#### Proposed model

We propose an approximate model built upon the two-compartment model. In two-compartment model, for each of the phases, e.g., association and dissociation phases, the bulk concentration in the first compartment is assumed constant and equal to its initial value. However, in our system, the finite duration input to the system results in plug-like passage of the transmitted ligands over the receiver surface as shown in Fig 1C, making it impractical to directly apply the two-compartment model.

Our strategy is to consider the whole process again as a two-phase process consisting of association and dissociation phases, and then, set the start time instants of association and dissociation phases to reflect the plug-like passage. We also define an effective plug length and effective plug concentration in order to elaborate the system within the framework of two-compartment model.

We start by modeling the propagation in the microfluidic convection-diffusion channel. Once we are done with the propagation, we incorporate the receiver reactions into the model with a modified two-compartment model.

Distance between transmitter and receiver *d* is taken as the distance of the center of the receiver to the entrance of the channel where transmission occurs:

$$\begin{array}{c} d=d_{rx}+l_{rx}/2. \end{array}$$ (23)

When the transmitter sends an impulse signal in the form of surface concentration, i.e., *N*/(*A*_*ch*_*N*_*A*_) = 1(*mol*/*m*^2^), neglecting the reaction at the receiver for the moment, the ligand concentration anywhere in the channel at any time can be written as [14]

$$\begin{array}{c} c_{imp}\left(x,t\right)=\frac{1}{\sqrt{4\pi Dt}}\exp\left(-\frac{{\left(x-ut\right)}^{2}}{4Dt}\right). \end{array}$$ (24)

The concentration function is Gaussian with variance $\sigma_{imp}^{2}=2Dt$, which is varying with time, making the subsequent calculations analytically untractable. Therefore, during the passage of the resulting ligand plug, we neglect the dispersion and assume that the variance is constant at the receiver location, making the plug a Gaussian function with variance $\sigma_{R}^{2}=2Dd/u$, moving at the flow velocity. Hence, end-to-end impulse response of the communication channel from the transmitter to the receiver location becomes

$$\begin{array}{c} c_{R}^{imp}\left(t\right)=\frac{1}{\sqrt{4\pi Dd/u}}\exp\left(-\frac{{\left(d-ut\right)}^{2}}{4Dd/u}\right). \end{array}$$ (25)

In this study, we assume a more practical signal, i.e., finite-length rectangular pulse, as the input. Accordingly, transmitter releases a total of *N* molecules at a constant rate *μ*_*T*_ for a specified pulse duration *T*_*p*_ uniformly through the entire surface of the channel inlet; thus, the transmitted signal can be written as

$$\begin{array}{c} s\left(t\right)=\frac{\mu_{T}}{A_{ch}N_{A}}\text{rect}\left(\frac{t}{T_{p}}-0.5\right), \end{array}$$ (26)

where rect(*t*) = 1 for −0.5 < *t* < 0.5 is the rectangular function, and *μ*_*T*_ = *N*/*T*_*p*_ is the transmission rate in *molecules*/*s*.

The convection-diffusion channel excluding the ligand-receptor reactions is a linear time-invariant (LTI) system; thus, the response to a rectangular pulse can be found via convolution, i.e.,

$$\begin{array}{c} c_{R}^{rect}\left(t\right)=s\left(t\right)*c_{R}^{imp}\left(t\right)=\frac{\mu_{T}}{2uA_{ch}N_{A}}\left[\text{erf}\left(\frac{ut-d}{2\sqrt{Dd/u}}\right)-\text{erf}\left(\frac{ut-uT_{p}-d}{2\sqrt{Dd/u}}\right)\right], \end{array}$$ (27)

where *N*_*A*_ is the Avogadro’s number.

We define the plug delay as the time of the passage of the peak ligand concentration through the center of the receiver, i.e.,

$$\begin{array}{c} t_{D}=d/u+T_{p}/2. \end{array}$$ (28)

To utilize the two-compartment model, we approximate the Gaussian ligand concentration pulse passing over the receiver as a finite duration pulse with a pulse length set to cover approximately 95–96% of the ligands, as done in [28]. This effective plug length is computed as

$$\begin{array}{c} w_{p}^{rect}=4\sigma_{R}+T_{p}u, \end{array}$$ (29)

where $\sigma_{R}=\sqrt{2Dd/u}$. Then the effective plug passage is given as

$$\begin{array}{c} \tau_{p}^{rect}=\frac{w_{p}^{rect}}{u}. \end{array}$$ (30)

Every point along the receiver surface is assumed to be exposed to a stationary concentration between a time window, whose end-points are marked by association and dissociation times. Taking the plug delay as the central point in time, we set the association time as

$$\begin{array}{c} t_{a}=t_{D}-\frac{\tau_{p}^{rect}}{2}, \end{array}$$ (31)

and the dissociation time is set to

$$\begin{array}{c} t_{d}=t_{D}+\frac{\tau_{p}^{rect}}{2}. \end{array}$$ (32)

Then, the effective plug concentration is calculated as the time-average of the concentration passing through the receiver surface between *t*_*a*_ and *t*_*d*_, i.e.,

$$\begin{array}{c} c_{avg}=\frac{1}{\tau_{p}^{rect}}\int_{t_{a}}^{t_{d}}c_{R}^{rect}\left(t\right)dt=\frac{1}{\tau_{p}^{rect}}\left(Q\left(t_{d}\right)-Q\left(t_{a}\right)\right), \end{array}$$ (33)

where *Q*(*t*) is given by

$$\begin{array}{ccc} Q(t) & = & \frac{1}{2u^{2}}\left[\left(ut-d\right)\text{erf}\left(\frac{ut-d}{2\sqrt{Dd/u}}\right)-\left(ut-uT_{p}-d\right)\text{erf}\left(\frac{ut-uT_{p}-d}{2\sqrt{Dd/u}}\right)\right]\\ & & +\sqrt{\frac{Dd}{\pi u^{5}}}\left(\frac{N_{m}}{A_{ch}}\right)\left[\exp\left(-\frac{{\left(ut-d\right)}^{2}}{4Dd/u}\right)-\exp\left(-\frac{{\left(ut-uT_{p}-d\right)}^{2}}{4Dd/u}\right)\right]. \end{array}$$ (34)

Given the definitions regarding the effective plug concept, the time course of mean number of bound receptors for a system where transmission starts at *t* = 0 obtained by the two-compartment model can be given by

$$\begin{array}{ccc} N_{R}^{*}\left(t\right) & = & N_{R,eq}^{*}\left(1-\frac{W_{0}\left(\alpha^{*}-\beta^{*}\left(t-t_{a}\right)\right)}{\alpha^{*}}\right)\left(\Theta \left[t-t_{a}\right]-\Theta \left[t-t_{d}\right]\right)\\ & & +\gamma^{*}W_{0}\left(-\frac{N_{R,0}}{\gamma^{*}}\exp\left(\frac{-k_{1}N_{R,0}-k_{T}^{*}k_{-1}\left(t-t_{d}\right)}{k_{1}\gamma^{*}}\right)\right)\Theta \left[t-t_{d}\right], \end{array}$$ (35)

where Θ[.] denotes the Heaviside step function, and the modified parameters are given as follows

$$\begin{array}{c} N_{R,eq}^{*}=\frac{c_{avg}}{c_{avg}+K_{D}}N_{R,max}, \end{array}$$ (36)

$$\begin{array}{c} \alpha^{*}=\frac{k_{1}c_{avg}N_{R,max}}{k_{-1}N_{R,max}+k_{T}^{*}\left(c_{avg}+K_{D}\right)}, \end{array}$$ (37)

$$\begin{array}{c} \beta^{*}=\frac{k_{1}c_{avg}+k_{-1}}{1+\frac{k_{-1}N_{R,max}}{k_{T}^{*}\left(c_{avg}+K_{D}\right)}}, \end{array}$$ (38)

$$\begin{array}{c} \gamma^{*}=\frac{k_{1}N_{R,max}+k_{T}^{*}}{k_{1}}, \end{array}$$ (39)

where the transport parameter is set to $k_{T}^{*}=k_{T}\times k$ to be optimized for our system.

#### Received pulse characteristics

In this section, based on the proposed model, we attempt to characterize the microfluidic MC channel by providing analytical expressions for three metrics: pulse delay, pulse amplitude, pulse width. A similar approach has been previously taken for diffusion-based MC channel in [29]. In the next section, we will compare our results to that obtained through finite element analysis numerical solutions in COMSOL Multiphysics for different system settings.

**Pulse delay**, different from the plug delay given in Eq (28), is defined as the time instant, at which the number of bound receptors reaches its peak value. In our model, the number of bound receptors given by Eq (35) is monotonically increasing for *t* ≤ *t*_*d*_, and monotonically decreasing for *t* > *t*_*d*_. Therefore, the pulse delay can be written as

$$\begin{array}{c} t_{pd}=t_{d}=t_{D}+\frac{\tau_{p}^{rect}}{2}=\frac{d}{u}+\frac{T_{p}+\tau_{p}^{rect}}{2}=\frac{d}{u}\left(1+\sqrt{\frac{8D}{ud}}\right)+T_{p}. \end{array}$$ (40)

Then, the **pulse amplitude**, as the peak value of the received signal, is given by

$$\begin{array}{c} N_{R,pa}=N_{R}^{*}\left(t_{pd}\right). \end{array}$$ (41)

Another important metric is the **pulse width**. As done in [29] for MC, we define it as the time interval, at which the pulse magnitude is greater than half of its peak value. Pulse width has implications for the achievable bandwidth as it determines the extent of intersymbol interference (ISI). Based on our model, pulse width is calculated as

$$\begin{array}{c} \tau_{pw}=\tau_{p}^{rect}-\frac{k_{1}\gamma^{*}\left(\ln\left(\frac{1}{2}\right)-\frac{N_{R,pa}}{2\gamma^{*}}\right)+k_{1}N_{R,pa}}{k_{T}k_{-1}}-\frac{1}{\beta^{*}}\left(\frac{\alpha^{*}N_{R,pa}}{2N_{R,eq}^{*}}-\ln\left(1-\frac{N_{R,pa}}{2N_{R,eq}^{*}}\right)\right), \end{array}$$ (42)

where $N_{R,eq}^{*}$, α*, β*, and γ* are given in Eqs (36)–(39).

The above analytical expressions for the received pulse delay, pulse amplitude and pulse width are of paramount importance for MC engineering, as they enable an optimization framework that can be utilized to optimize the overall system from ICT perspective, and help develop advanced communication schemes, such as optimum detection methods and modulation techniques.

## Results and discussions

In this section, we first estimate the transport parameter $k_{T}^{*}=k\times k_{T}$, and then, evaluate the acuracy of the proposed analytical model by comparing the calculations under different conditions to the results obtained via finite element analyses in COMSOL Multiphysics. The default values for the system parameters used in the analyses are listed in Table 1.

**Table 1:** Default values of simulation parameters.

Microfluidic channel height (*h*_*ch*_)	20 *μ*m
Microfluidic channel width (*w*_*ch*_)	20 *μ*m
Rete of ligand transmission (*N*_*m*_)	1 × 10^9^ 1/s
Transmitted pulse length (*T*_*p*_)	0.5 s
Distance to the front-end of the receiver (*d*_*rx*_)	15 mm
Max flow velocity (*u*)	50 *μ*m/s
Intrinsic diffusion coefficient of ligands (*D*_0_)	1 × 10^−10^ m^2^/s
Binding rate (*k*_1_)	1 × 10^2^ m^3^/(mol⋅s)
Unbinding rate (*k*_−1_)	1 × 10^−2^ 1/s
Surface concentration of receptors at the receiver (*ρ*_*SR*_)	1 × 10^−8^ mol/m^2^
Receiver length along the x-axis (*l*_*rx*_)	20 *μ*m

**Fig 2:**
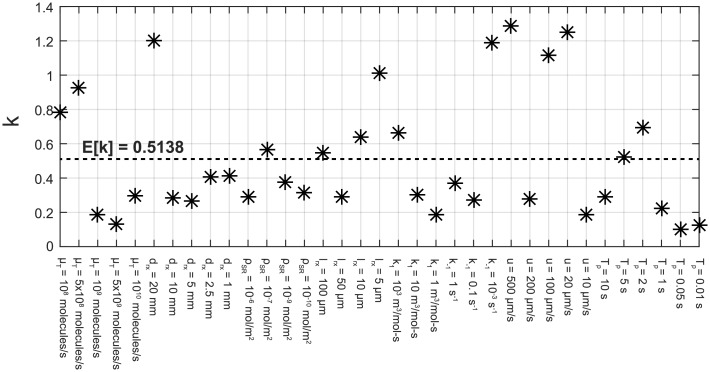
Optimal values of free parameter *k* obtained with varying system parameters.

To find the optimal value of the free parameter *k*, we perform nonlinear least square estimation in MATLAB using Levenberg-Marquardt optimization algorithm. The curve fitting is conducted on the numerical results obtained via COMSOL for 35 different scenarios, in each of which we vary only one parameter from its default setting at a time. The results of the optimization with the corresponding system parameters are presented in Fig 2. The mean and standard deviation of the obtained data are 0.5138 and 0.3616, respectively. Setting *k* to its mean, we rewrite the transport parameter optimized for the communication scenario as

$$\begin{array}{c} k_{T}^{*}=0.5138\times k_{T}=0.7553\left(\frac{1-{\left(d_{rx}/\left(d_{rx}+l_{rx}\right)\right)}^{2/3}}{1-d_{rx}/\left(d_{rx}+l_{rx}\right)}\right)\sqrt[{3}]{\frac{D^{2}F}{h_{ch}^{2}w_{ch}\left(d_{rx}+l_{rx}\right)}}. \end{array}$$ (43)

**Fig 3:**
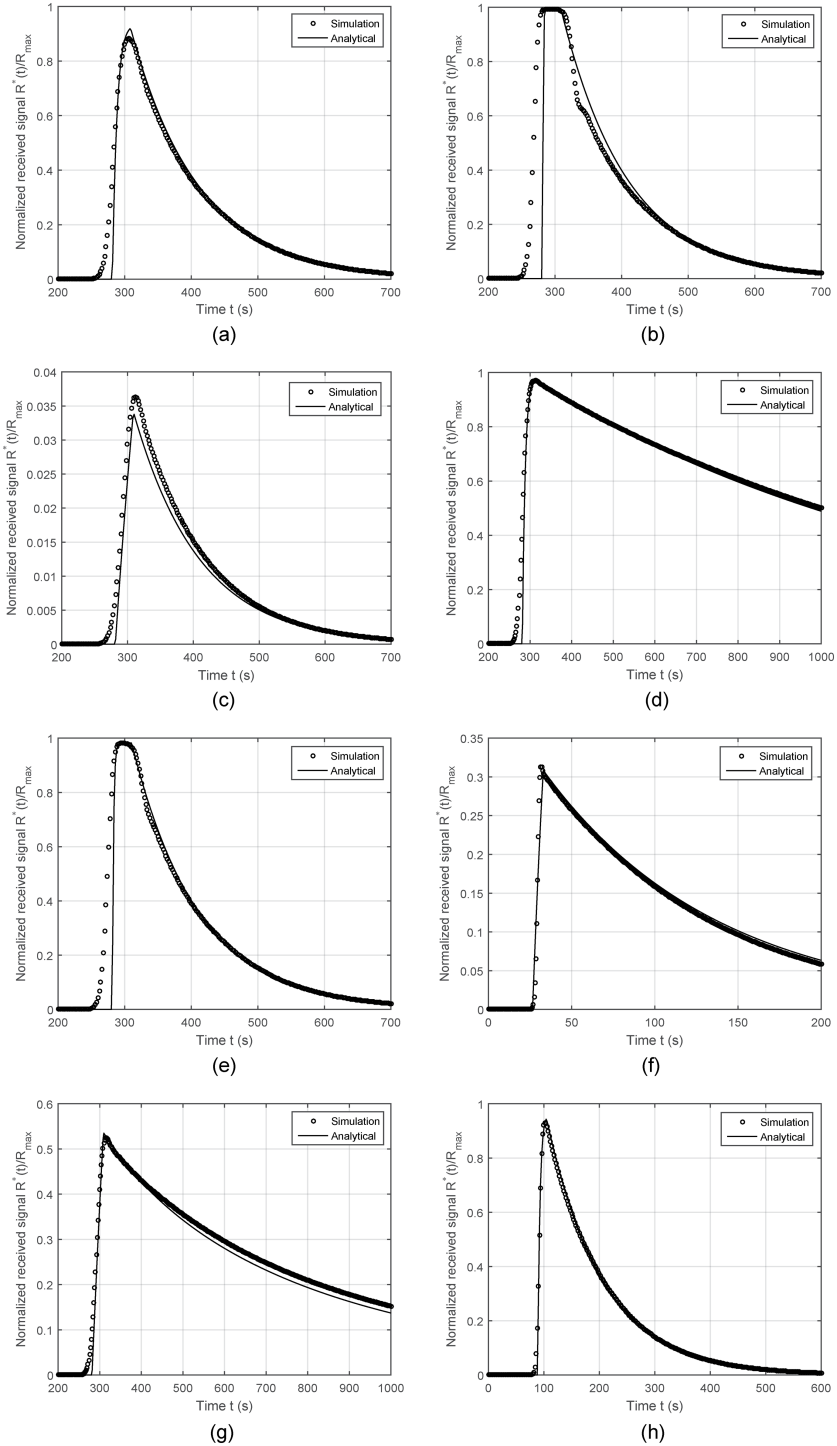
Time course of the number of bound receptors normalized by the total number of receptors for varying system parameters. In each analysis, only one parameter is changed from its default value given in Table 1. (A) Default setting. (B) *μ*_*T*_ = 10^10^ molecules/s. (C) *k*_1_ = 1 m^3^/mol⋅s. (D) *k*_−1_ = 10^−3^ s^−1^. (E) *T*_*p*_ = 5 s. (F) *u* = 500 *μ*s. (G) *ρ*_*SR*_ = 10^−6^ mol/m^2^. (H) *d*_*rx*_ = 5 mm.

Using the optimized transport parameter, we compare the time course of normalized number of bound receptors obtained via Eq (35) under different scenarios to the numerical results of COMSOL experiments. The results are presented in Fig 3. Clearly, our analytical model approximates quite well the numerical solution, justifying the accuracy of the model.

**Fig 4:**
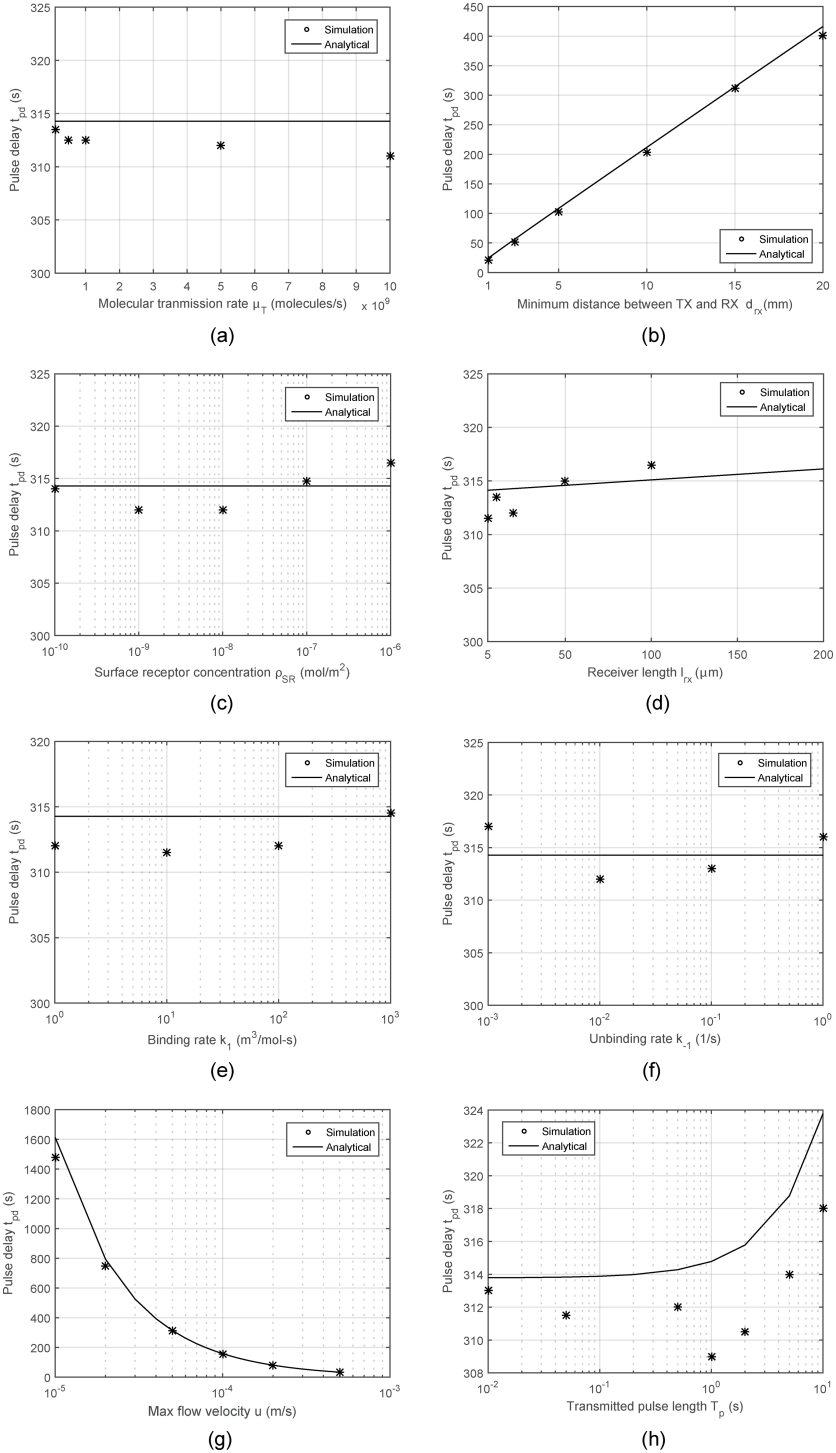
Received pulse delay *t*_*pd*_ with varying system parameters.

In the second part, we analyze the capability of our model to reflect the characteristics of the received signal under various conditions, this time, using the metrics defined in the previous section. Accordingly, the pulse delay obtained via Eq (40) is compared to the numerically computed results in Fig 4. As is seen, the simple expression given in Eq (40) is quite accurate in approximating the exact results and following the trends with varying parameter values. It is worth noting that the received pulse delay is not affected by the molecular transmission rate, surface receptor concentration, and the binding/unbinding rates of the ligand-receptor pairs. As expected, it is most affected by the minimum TX-RX distance *d*_*rx*_ and flow velocity *u*.

**Fig 5:**
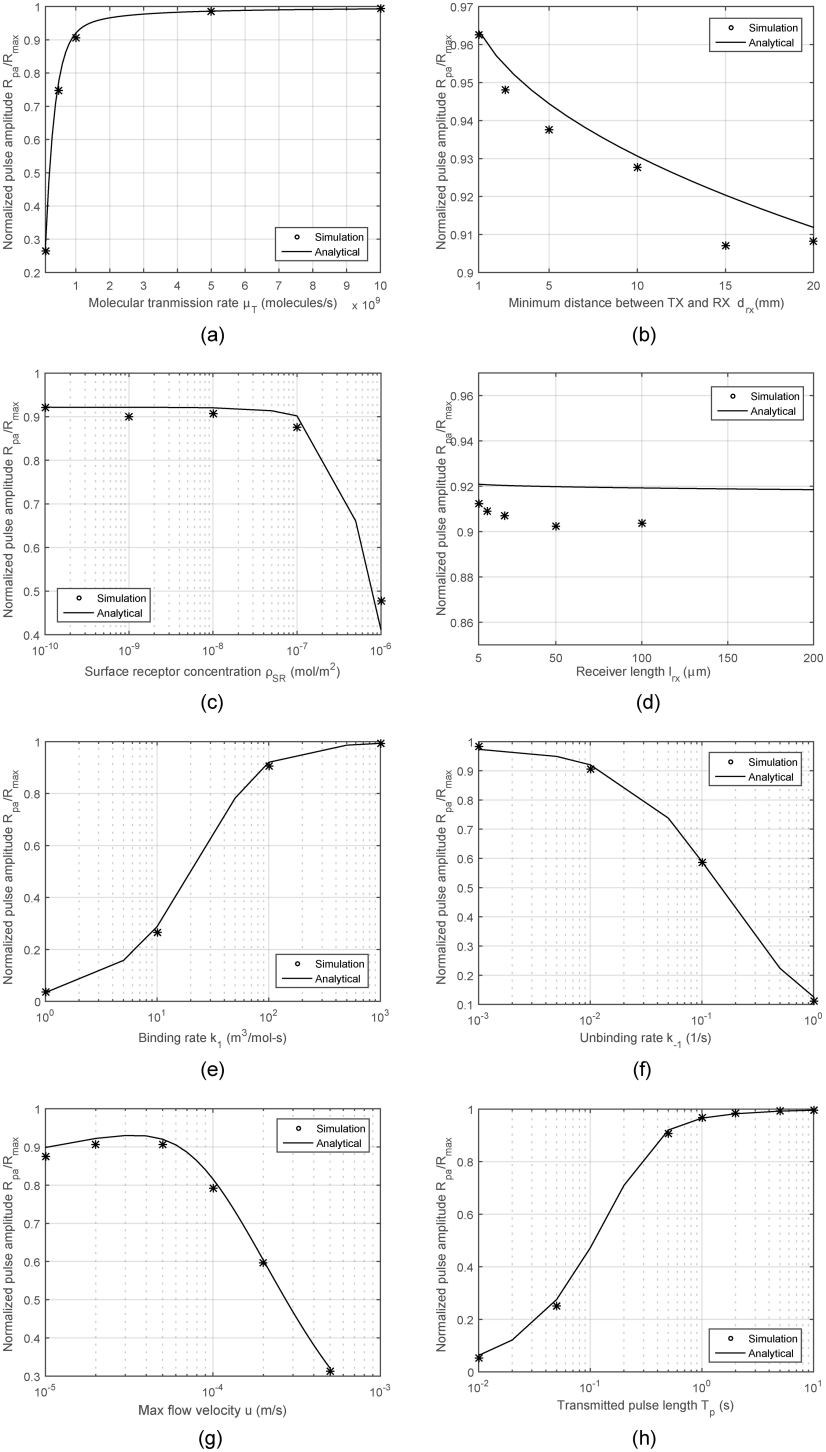
Normalized received pulse amplitude *R*_*pa*_/*R*_*max*_ with varying system parameters.

The analyses are repeated for the normalized pulse amplitude, which is the ratio of the peak number of bound receptors to the total number of receptors. Expected pulse amplitude is quite important for the communication system design, as it determines the received SNR. The results obtained via Eq (41) are compared to numerical results in Fig 5. The parameters range between values corresponding to sparsely occupied and saturated receiver. The results reveal that almost all parameters have substantial effect on the pulse amplitude, and the exact numerical results and the corresponding trends are well approximated by the proposed analytical model, no matter whether the receiver is saturated or sparsely occupied. One interesting result observed by both numerical and analytical calculations is obtained for varying flow velocity. As shown in Fig 4G, given the fixed molecular transmission rate, the pulse amplitude is decreasing when the flow velocity is higher or lower than a certain optimal value. This is because for low velocities, the degree of attenuation through dispersion until the plug arrives at the receiver location increases, and for high velocities, the time duration, at which the ligand plug and receiver are in contact decreases, leading to a lower number of bound receptors. The same trend was also observed and discussed in detail in [30–32].

**Fig 6:**
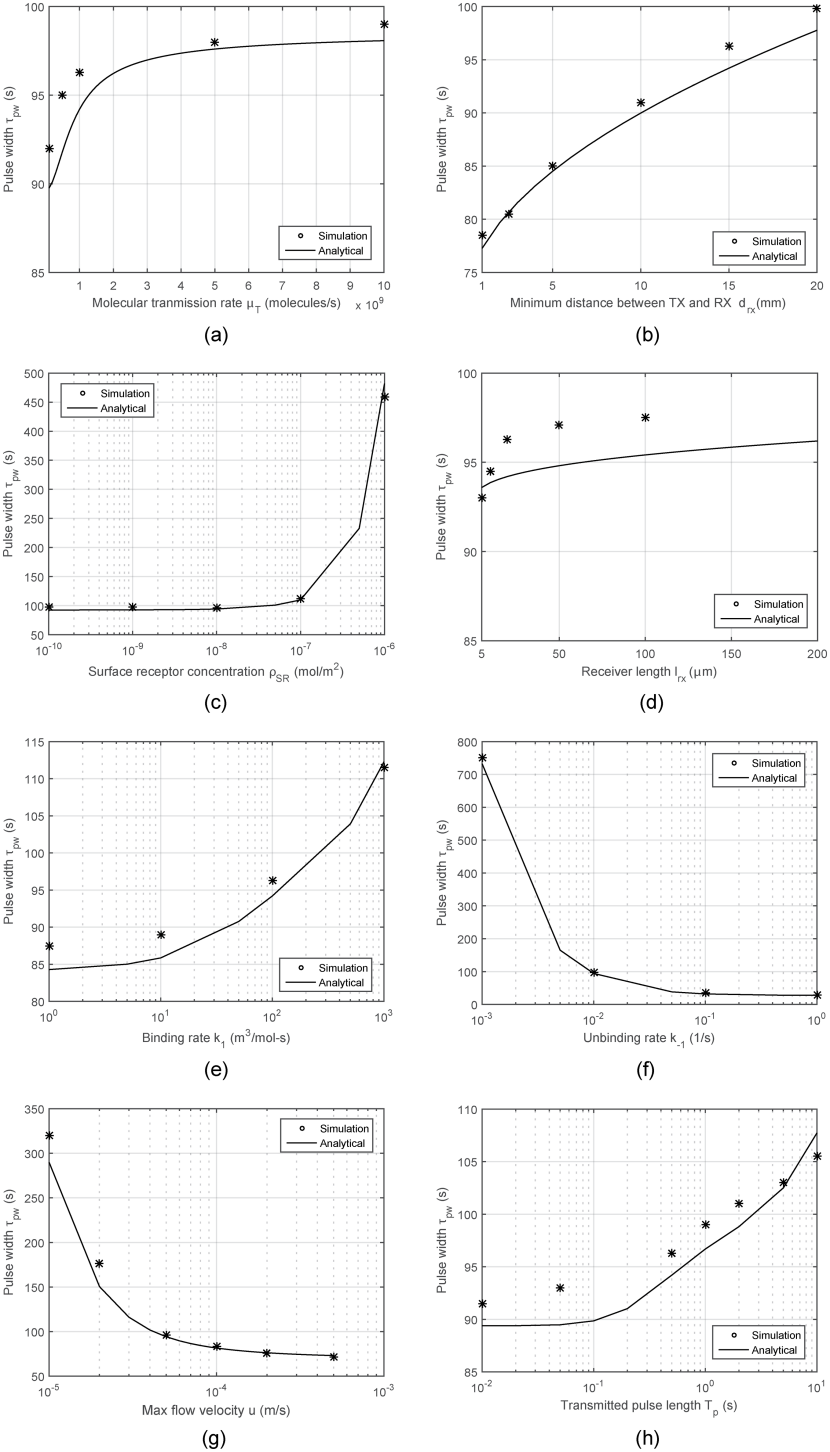
Received pulse width *τ*_*pw*_ with varying system parameters.

The third set of analyses are conducted for the pulse width, which is an important parameter since it is directly linked to the extent of ISI and achievable bandwidth. Given a fixed symbol duration, ISI increases with pulse width. The analytical results compared to the numerical calculations are presented in Fig 6. The proposed model quite well reflects the characteristic trends observed under varying conditions. The results reveal that the unbinding rate of the employed ligand-receptor pair is the most critical parameter to adjust the received signal pulse width.

Our model, which is quite simple, practical, not requiring the computationally expensive numerical methods, is able to accurately capture the design tradeoffs, and could be used to design efficient and reliable microfluidic MC system before the final implementation.

Finally, we would like to discuss about an alternative communication scheme that can be targeted to improve the performance of the microfluidic MC with surface-based receivers. The performance could be substantially improved if multiple types of ligand-receptor pairs with different binding characteristics, are employed in the communication system [4]. This way, the transmitter can employ molecular division multiplexing, similar to the code division multiplexing in conventional EM communication systems, to boost the communication rate by simultaneously transfering multiple messages in the same channel without causing significant interference. This can be reliazed in two ways. (i) Employing multiple receiver antennas, e.g., surface-based biosensors, with each having different kind of receptors that bear affinity to different kinds of ligands. In this scheme, the receiver can process the output of each antenna separately with minimum interference. (ii) Employing multiple types of receptors, at a single receiver antenna, corresponding to different kinds of ligands. In this scheme, the output of different kinds of receptors are superposed at the receiver output; therefore, this scheme requires employing more complex signal processing to discriminate the contributions of different messages conveyed through different ligands. Fortunately, there are some studies proposing frequency domain detection techniques to exploit the diversity in the critical frequency of ligand-receptor binding noise.

## Conclusion

In this paper, we develop an analytical model for microfluidic MC systems with surface-based receivers equipped with ligand receptors to approximate the time course of the number of bound receptors. The model is based on the two-compartment model of convection-diffusion-reaction systems, which is tailored to the peculiarities of the communication system. The comparison of the analytical model-based results with the numerical results obtained by solving the exact model in COMSOL proves the accuracy of the developed model, which is quite successful in capturing the nonlinearities of the system. We also provide analytical expression for received pulse width, pulse amplitude and pulse delay to provide a framework that would help optimize the microfluidic systems from communication theoretical perspective.
